# Parasyphilis

**Published:** 1905-05-27

**Authors:** 


### PARASYPHILIS.

It would appear that the dependence of tabes dorsalis and general paralysis upon a basis of syphilis is not so generally accepted by French physicians as it is in this country, for Professor Fournier has felt himself called upon to defend his conception of general paralysis as a parasyphilitic lesion in a communication to the Academy of Medicine.[1] In the course of a spirited and conclusive reply to the opponents of his thesis, he cites the remarkable case of the seven glass-blowers who were all infected at the same time by a contaminated mouthpiece, and all developed labial chancres. An attempt was made to trace them ten years later, when five of the seven were discovered. Of these five, one was in good health, two were tabetic, and the remaining two were general paralytics. Professor Fournier very fairly refuses to believe that in the face of such a series any man of sense will invoke coincidence to explain away the sequel of the syphilitic infection.

Amongst other corroborative detail the author quotes the statement of Babinski to the effect that there is no case on record of a man having contracted syphilis subsequent to the appearance in him of an Argyll-Robertson pupil, which is as weighty as negative evidence can be. Professor Fournier protests against an arbitrary definition of the pathological anatomy of syphilis, to be followed by the rigid exclusion from the syphilitic category of any lesion which does not conform to the arbitrary type. Besides tabes and general paralysis, which are the two best-known lesions of the reputed parasyphilitic type, the author includes leucoplakia. This, of course, bears no anatomical resemblance to the accepted lesions of syphilis, yet he is of opinion that not less than 80 or 90 per cent. of cases of this disease are parasyphilitic, while some authors follow Landouzy in claiming syphilis as the basis of them all, averring that "the cases where specific antecedents are lacking are cases of ignored or unrecognised syphilis." "I need not dilate," continues Professor Fournier, "upon the clinical importance of parasyphilis, since it is responsible for such manifestations as, in the case of the acquired disease, tabes dorsalis, general paralysis, hystero-syphilis and leucoplakia, and, in the case of the inherited form, dystrophic lesions of every kind, malformations, rachitism, and hydrocephalus, not to mention juvenile tabes and juvenile general paralysis."

While upon the subject of general paralysis we may briefly refer to a recent contribution by Dr. Dana,[2] in which he claims that general paralytics are susceptible to treatment in the very earliest stages of the disease. He cites a number of cases where, in his opinion, general paralysis was clearly threatening, cases which under the influence of thorough mercurial treatment by hypodermic injection, combined with the general treatment suitable to early mental diseases, became either stationary or well. In some of these an Argyll-Robertson pupil already established is reported to have become active to light, and the restoration to health has remained complete for several years. Dr. Dana is no optimist as regards general paralysis in general, and believes it to be incurable when once established, as indeed it must be by reason of the organic brain-changes which accompany it. But many alienists hold that disorders of function may result in changes of structure, and it is something to find any responsible person holding out a hope of relief in this most inveterate of diseases. The great difficulty of the problem lies in the doubt concerning the justice of the diagnosis in these very early cases, and the bulk of Dr. Dana's audience professed a not unnatural scepticism on this head.

[1] Revue de Thérap. Medico-Chirurg., May 1, 1905. [2] Journ. of Amer. Med. Ass., May 6, 1905.

